# PowerPlex^®^ Fusion 6C System: evaluation study for analysis of casework and database samples

**DOI:** 10.3325/cmj.2017.58.26

**Published:** 2017-02

**Authors:** Selena Cisana, Nicoletta Cerri, Alessandro Bosetti, Andrea Verzeletti, Venusia Cortellini

**Affiliations:** 1 University of Brescia - Department of Medical and Surgical Specialties, Radiological Sciences and Public Health - Forensic Medicine Unit, Brescia, Italy; 2 Promega Italia s.r.l. Via Decembrio 28, 20137 Milano, Italy

## Abstract

**Aim:**

To report on the successful analysis of amplicons obtained with PowerPlex^®^ Fusion 6C System, a highly robust 27-plex genotyping kit developed for human identification laboratories, on the Applied Biosystems^®^ 3500 Genetic Analyzer.

**Method:**

We performed characterization and evaluation studies following the Scientific Working Group on DNA Analysis Methods (SWGDAM) validation guidelines, examining several critical areas of kit performance. We report the results of sensitivity, robustness, heterozygous peak height ratio, precision, concordance, caseworks, and mixture interpretations. We tested sensitivity, using serial dilutions of control DNA.

**Results:**

The minimum amount of input DNA resulting in a full profile was 125 pg. Inhibition, inducted by urea, showed a progressively fragmentation of DNA and a full profile was obtained until 1M of inhibitor factor. To test the profile quality, casework samples were extracted with different extraction methods: Chelex®100, QIAmp DNA Micro Kit and Phenol-Chloroform extraction. The results demonstrated that extraction chemistries do not have affect on amplification performance. Concordance check was performed by typing some casework samples and comparing the typing results with those obtained with other available kits. Thus, concordance was expected and supported by the data.

**Conclusion:**

Reliable DNA typing results can be obtained using this new kit, demonstrating its effectiveness and utility in forensic analysis.

The original Combined DNA Index System (CODIS) database consisting of 13 core short tandem repeat (STR) loci (CSF1PO, D3S1358, D5S818, D7S820, D8S1179, D13S317, D16S539, D18S51, D21S11, FGA, TH01, TPOX, and vWA) has been successful for matching suspects with evidence. However some situations require inclusion of more loci and increased discrimination, such as mixture interpretation and degradation (1). The PowerPlex^®^ Fusion 6C System is a 27-locus multiplex for human identification applications including forensic analysis, relationship testing and research use. This six-color system allows co-amplification and fluorescent detection of the 18 autosomal loci in the expanded CODIS core loci (CSF1PO, FGA, TH01, vWA, D1S1656, D2S1338, D2S441, D3S1358, D5S818, D7S820, D8S1179, D10S1248, D12S391, D13S317, D16S539, D18S51, D19S433 and D21S11) as well as Amelogenin and DYS391 for gender determination. The Penta D, Penta E, D22S1045, TPOX, SE33 loci and the two rapidly mutating Y-STRs, DYS570 and DYS576 loci, are also included to increase discrimination, satisfying both CODIS and European Standard Set (ESS) recommendations (2,3). The new loci may also aid in determining the number of contributors to a mixture (4).

This kit alleviates a significant bottleneck in genotype throughput by eliminating the need for more amplifications to obtain complete profiles for a particular sample through a single tube analysis. The PowerPlex^®^ Fusion 6C System is compatible with the Applied Biosystems^®^ 3500 Genetic Analyzer as reported in the technical manual (5), but a validation should be performed.

This paper describes our validation studies conducted with PowerPlex^®^ Fusion 6C System, based on internal validation criteria in accordance with guidelines issued by the Scientific Working Group on DNA Analysis Methods (SWGDAM) (6). We performed sensitivity, inhibited and mixture studies using control DNA samples and we evaluated the data obtained from a set of casework samples previously typed. The results of this validation showed that PowerPlex^®^ Fusion 6C System, producing accurate and reproducible STR profiles, improved the internal laboratory procedures.

## MATERIALS AND METHODS

### Sensitivity

2800M Control DNA (Promega, Madison, Wisconsin, USA) was serially diluted in 2-fold increments to give the following amounts when added at 25 µL per PCR: 1.0 ng, 500 pg, 250 pg, 125 pg, 62.5 pg, and 31.25 pg total per reaction. Two replicate PCRs were prepared and PCR amplifications were performed according to manufacturer’s protocol. For allele designation and peak height ratio determination a 175 RFU threshold was used for data.

### Inhibited model samples

One model of PCR inhibition, urea, was used to represent inhibitory compounds; the inhibitor stock solution was prepared as follows: urea (Panreac Quimica S.L.U., Barcelona, Espana) 10M in water. PCR samples were prepared with 1.0 ng of male 2800M control DNA containing a range of inhibitor concentrations: 0M (no inhibition), 1M, 2M, and 5M of urea. Each condition was tested in duplicate PCRs.

### DNA mixture study

In forensic caseworks the evidence often contains a mixture DNA from more than one individual. These mixtures can be very challenging to analyze and interpret. For improved mixture interpretation, two rapidly-mutating Y-STR loci, DYS570 and DYS576, are included in PowerPlex^®^ Fusion 6C System. To test the performance of this kit with DNA mixture samples, a series of mixtures of two different human genomic DNAs (male 2800M and female 9947A, Promega) was made in different mixing ratios. Mixtures were formulated to always give 1.0 ng of total DNA per 25 µL volume, as manufacturer’s recommended, with ratios of minor to major DNAs being 1:1, 1:5 and 1:10 and vice versa. For example, the 1:5 mixture contained 167 pg minor and 833 pg major DNA per reaction. Each mixture samples was tested in two PCRs and amplified according to manufacturer’s protocol.

### Forensic caseworks

In order to evaluate the ability to obtain reliable results, some forensic case samples were amplified using PowerPlex^®^ Fusion 6C System. The consistency of typing results was evaluated considering previously typing through already validated kits such as AmpF*l*STR^®^ Identifiler Plus (7), AmpF*l*STR^®^ NGM PCR kits (Life Technologies, Carlsbad, CA, USA), PowerPlex^®^ 16 HS System (8) or PowerPlex^®^ ESX 17 System (9) (Promega, Madison, WI, USA). DNA concentration was determined by measuring the absorbance of the sample at 260 nm, using the spectrophotometer NanoDrop 1000 (ThermoScientific, Waltham, Massachusetts, USA). All samples were amplified following manufacturer's protocols.

### Compatibility with different DNA extraction chemistries

Tested samples were extracted from a variety of sources by Chelex^®^100 (BIORAD, Richmond, CA, USA) (10), QIAamp DNA Micro Kit (Qiagen, GmbH, Hilden, Germany) (11) and Phenol-Chloroform extraction (12).

The detection was performed on an Applied Biosystems® 3500 Series Genetic Analyzer and data were analyzed with GeneMapper® ID-X software.

### Sizing precision

Sizing precision is fundamental to an accurate genotyping. The injection of the internal lane standard is the basis of an STR-typing system’s precision and essential to ensure genotyping accuracy. Sizing precision of the WEN ILS 500 size standard was evaluated by comparing the size of every allele in the allelic ladder over the course of 16 ladder injections during a run on Applied Biosystems^®^ 3500 Genetic Analyzer. The size of each allele was determined with GeneMapper^®^ ID-X v1.4 using the local Southern method. The size was retrieved from the genotypes’ tables and introduced into Excel spreadsheets to calculate the average and a standard deviation for each allele was plotted against the allele size.

### Sample electrophoresis and data analysis

Following thermal cycling, PCR products were prepared for capillary electrophoresis (CE) by combining 9.5 µL of Hi-DiTM Formamide, 0.5 µL of WEN ILS 500 and 1.0 µL of sample and performed on the 3500 Genetic Analyzer according to manufacturer’s manual. Genotyping was performed with GeneMapper® ID-X v1.4 software, using the provided panel and bin files. For allele designation and peak height ratio determination a 175 RFU threshold was used. In a forensic setting peaks are considered valid for use in statistical analysis only above this threshold and it is coherent with our observation of peak height ratios across multiple loci in dilution series of DNA amplified in replicate.

## RESULTS

### Sensitivity

**Figure 1:**
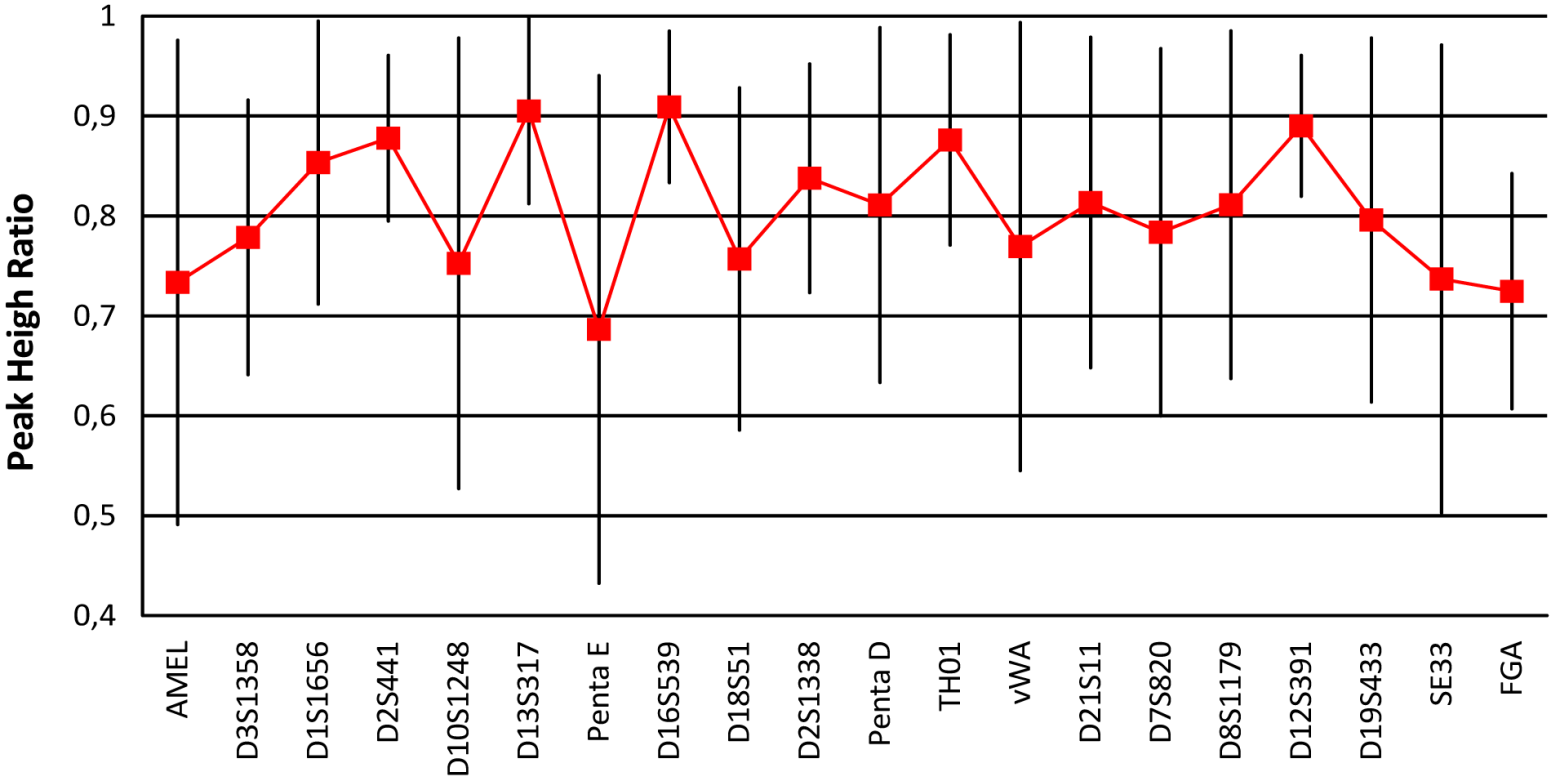
Peak height ratio analysis. The graph displays the range of peak height ratio for serially dilution in two replicate polymerase chain reactions (PCRs) for each locus. The mean of peak height ratio are also shown by red plot. The homozygous peaks and short tandem repeats (STR) on the Y-chromosome (Y-STR) were excluded.

We examined the sensitivity of the PowerPlex^®^ Fusion 6C System using serially dilution from 1 ng to 31.25 pg of 2800M Control DNA. Full allele profiles were obtained for 2800M at and above level of 125 pg, below which stochastic dropout of alleles was observed and partial profiles could be observed even at 62.5 pg and 31.25 pg of template DNA. For 2800M, the mean allele counts for the lowest DNA level tested were 17 alleles out of 47 possible, corresponding to the 36.2% of a full profile (Supplemental Figure 1(supplementary Figure 1)). For peak height ratio (PHR) determination loci with homozygous peaks and Y-STR’s were excluded from peak height ratio calculations, Amelogenin was also included. When drop out occurred, those loci were also excluded from peak height ratio calculations (Figure 1). The measurements for peak height were retrieved from the genotypes’ tables and introduced into Excel spreadsheets. The mean percentage heterozygous peak height ratio varied from a low 0.69 (SD 0.40) at locus Penta E to a high of 0.91 (SD 0.13) at locus D13S317. At least 95% of the observations showed PHR higher than 70% including the gender marker.

### Inhibited model samples

**Figure 2:**
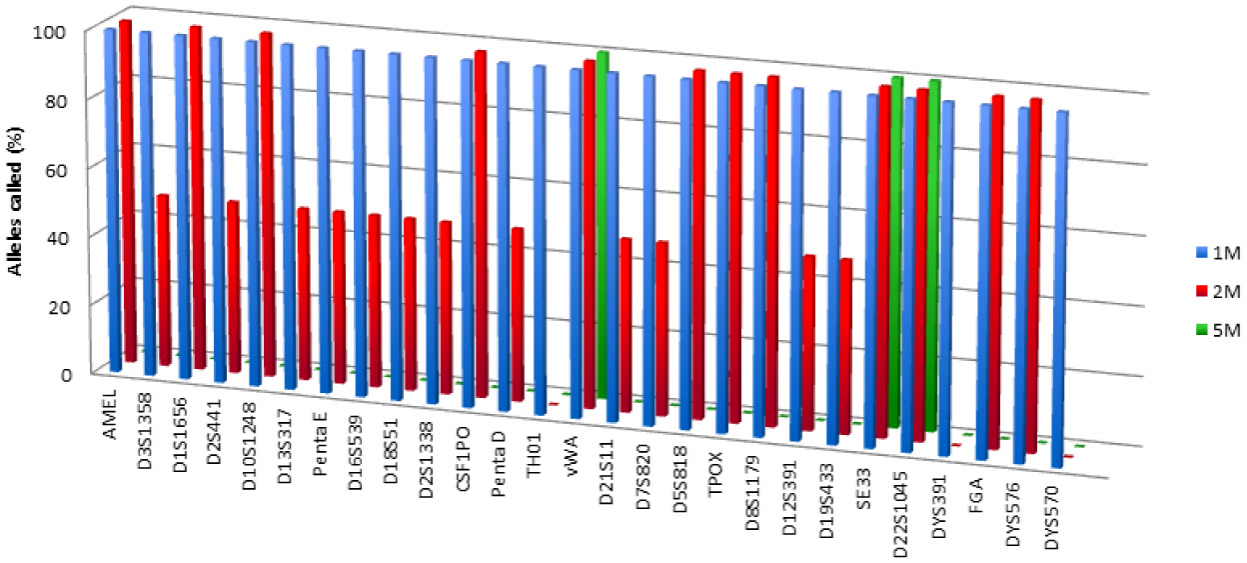
Different effect of inhibitor on amplification efficiency for each locus. The graph shows the different percentage of allele detected for each locus in different inhibited samples (1M, 2M, and 5M of urea). The untreated control “0M” gave full alleles (not included).

The robustness was tested using model systems for PCR inhibition. The effects of DNA inhibition on the amplification efficiency resulted in a progressively fractionated DNA. A graphical representation is shown in Supplemental Figure 2(supplementary figure 2). All replicates of the untreated controls and of the “1M” samples gave full profile. Partial profiles could be observed for “2M” samples and only a few alleles were obtained with the more highly inhibited samples, as reported in Table 1. The results are probably due to different effect of inhibitor on amplification efficiency for each primer pair (Figure 2).

**Table 1:** Amplification efficiency with increasing of urea concentration*

Sample	Count	%Full
0 M	47	100
1 M	47	100
2 M	31	66
5 M	5	11

*In “Sample” column are indicated the inhibitor concentrations. The untreated control is designed as “0 M”. “Count” column is the mean number of allele detected, and “%Full” column indicates the approximate percentage of a full profile for the DNA that was represented by the mean allele counts. The full profile contained 47 alleles total.

### DNA mixture study

**Figure 3:**
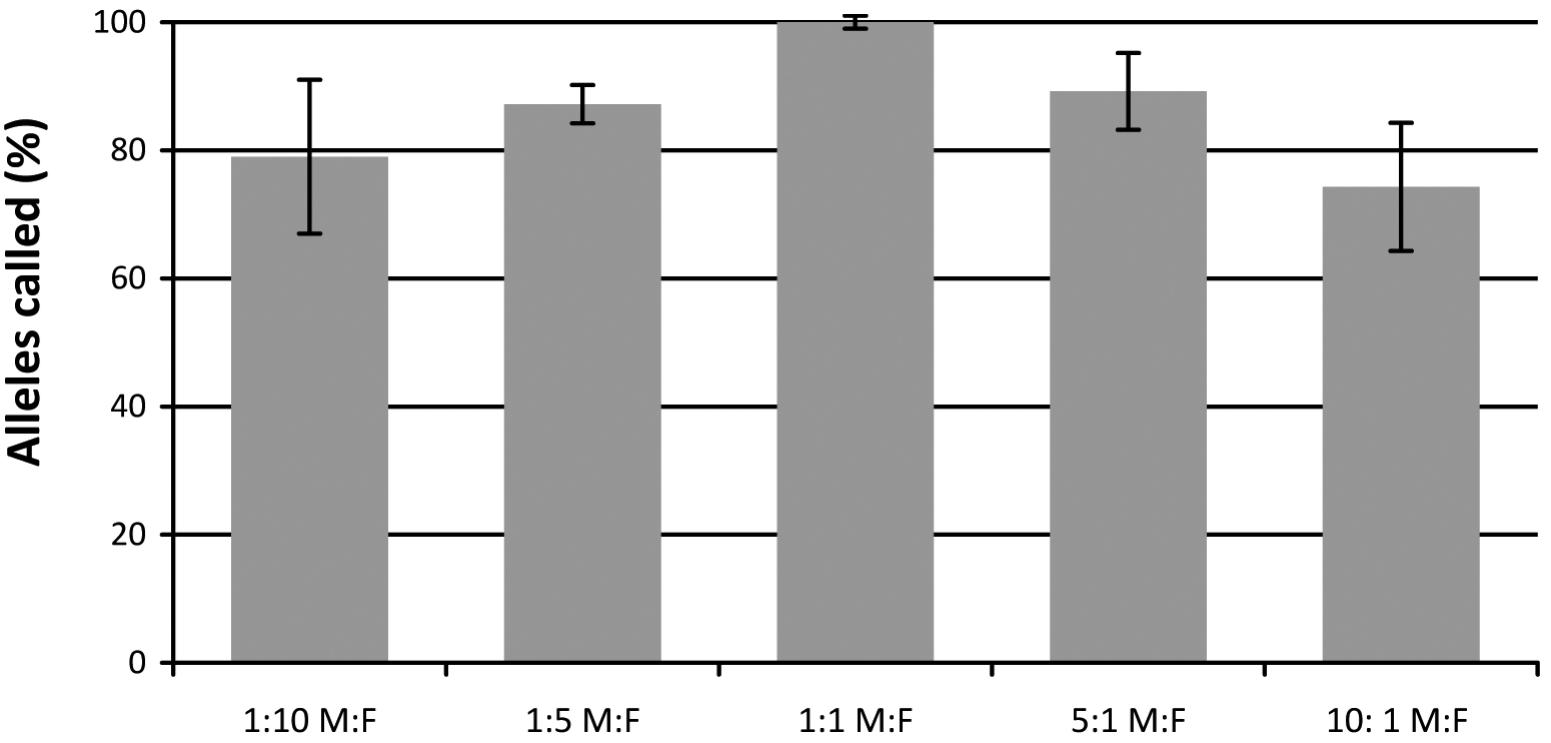
DNA mixture study. The average percentage of the alleles detected at a given mixture ratio is shown. Error bars represent the standard deviation of replicate results.

A two-known source DNA mixture was created at various ratios and amplified using the recommended protocol, to always give 1.0 ng of total DNA per 25 µL volume. Amplification products were detected using 175 RFU threshold. Based on peak height ratio assessments we confirmed the ratios for major:minor contributors to the formulated mixture. Following the relevant features for the assessment of mixed DNA profiles reported in literature (13), which described the criteria for identification of mixed specimens, all the alleles of the minor contributor were detected in all replicate PCRs of the 1:1, 1:5 and 5:1 mixtures (Figure 3). Results for the higher mixing ratio samples (1:10 and 10:1) reflect the stochastic nature of amplifying low input DNA levels. The detection limit of the minor component is about 10%. In 1:10 mixture (with major female DNA), which represents typical case situations, it was even possible to detect the presence of male DNA thanks to amplification of the Y-STR markers. A representative electropherogram is included in Supplemental Figure 3(supplementary figure 3).

### Forensic caseworks

To test the reliability of PowerPlex^®^ Fusion 6C System typing results we amplified 22 forensic case DNA samples, from our own collections (Table 2). Furthermore, as DNA databases are expanding, reproducibility between STR kits is increasingly important to ensure the accuracy of data generated with newer kits and maintain the relevance of data generated with older ones. The SWGDAM guidelines report that results from a new kit studies should be compared to the previous results of known samples to ensure concordance. Profile quality and sensitivity were not compared, since sampling methodology and kit chemistry are different. Instead, concordance evaluations are important to determine if there are any “null alleles” present in a data set. Depending on the STR kit configuration the placement of the markers can vary from kit to kit because the primer sequences were designed to amplify different product sizes. This allows concordance testing to be performed to compare results obtained between different kits. The results of this study were compared with genotypes previously generated with other kits (AmpFlSTR^®^ Identifiler Plus, AmpFlSTR^®^ NGM, PowerPlex^®^ 16 HS System or PowerPlex^®^ ESX 17 System). For all samples amplified with PowerPlex^®^ Fusion 6C System, concordance among the systems was observed and the typing reliability of the new primer sets has been validated. While not an indication of discordance, the PowerPlex^®^ Fusion 6C System kit resulted in more loci designation. This new kit includes 27 STR markers and increases discrimination, especially in the tested mixture samples, where a higher number of allele was detected.

**Table 2:** Case-type samples evaluated with the PowerPlex^®^ Fusion 6C System and comparison of case-type samples previously genotyped with other kits (AmpFlSTR^®^ Identifiler Plus, AmpFlSTR^®^ NGM PCR kits, PowerPlex^®^ 16 HS System or PowerPlex^®^ ESX 17 System)

Sample*	PowerPlex® Fusion 6C Alleles number	Previous typing kit	Alleles number	Concordance
Buccal swab	47 full profile	AmpFlSTR® NGM PCR	37	YES
Blood sample	37 full profile	AmpFlSTR® Identifiler® Plus	25	YES
Blood sample	38 full profile	PowerPlex® 16 HS System	3	YES
Swab of glove	24	PowerPlex® 16 HS System	27	YES
Swab of glove	17	PowerPlex® 16 HS System	18	YES
Swab of glove	24	PowerPlex® 16 HS System	13	YES
Swab of glove	7	PowerPlex® 16 HS System	0	N/A
Swab of glasses	11	PowerPlex® ESX 17 System	27	YES
Swab of glasses	9	PowerPlex® ESX 17 System	30	YES
Swab of mask	42	PowerPlex® ESX 17 System	13	YES
Swab of mask	20	PowerPlex® ESX 17 System	13	YES
Swab of mask	0	PowerPlex® ESX 17 System	0	N/A
Swab of neckband	16	PowerPlex® ESX 17 System	29	YES
Human tissue	45 full profile	AmpFlSTR® Identifiler® Plus	27	YES
Human tissue	8	AmpFlSTR® Identifiler® Plus	19	YES
Blood stain (cloth)	47 full profile	AmpFlSTR® Identifiler® Plus	29	YES
Blood stain (cloth)	45 full profile	AmpFlSTR® Identifiler® Plus	29	YES
Blood stain (cloth)	46 full profile	AmpFlSTR® Identifiler® Plus	30	YES
Case-type mixtures	69	AmpFlSTR® Identifiler® Plus	43	YES
Case-type mixtures	48	AmpFlSTR® Identifiler® Plus	40	YES
Case-type mixtures	70	AmpFlSTR® Identifiler® Plus	43	YES
Case-type mixtures	67	AmpFlSTR® NGM PCR	53	YES

*For each sample, the total number of alleles called with each kit is shown. Where loci had overlap between kits, they were examined for concordance.

### Compatibility with DNA extraction chemistries

Tested samples extracted with different analytical methods (Chelex^®^100, QIAamp DNA Micro Kit and Phenol-Chloroform extraction), provided profiles in which quality was comparable, confirming that different extraction chemistries do not adversely affect the amplification.

### Sizing precision

The sizing precision is important for STR fragment analysis since some alleles differ by only 1 base in size. Key performance requirements for the use of capillary electrophoresis (CE) for separation and detection of amplified fragments are accurate sizing of fragment over the fractionation range of the STR multiplex amplification system, single-base resolution over the fractionation range, and high run-to-run precision to enable comparison of mobility of alleles in the allelic lad­der to sample’s alleles. Size precision of the WEN ILS 500 size standard was evaluated by comparing the size of each allele in the allelic ladder over the course of 16 ladder injections during a run on the Applied Biosystems® 3500 Genetic Analyzer. The size of each allele was determined with GeneMapper^®^ ID-X v1.4 using the local Southern method.

**Figure 4:**
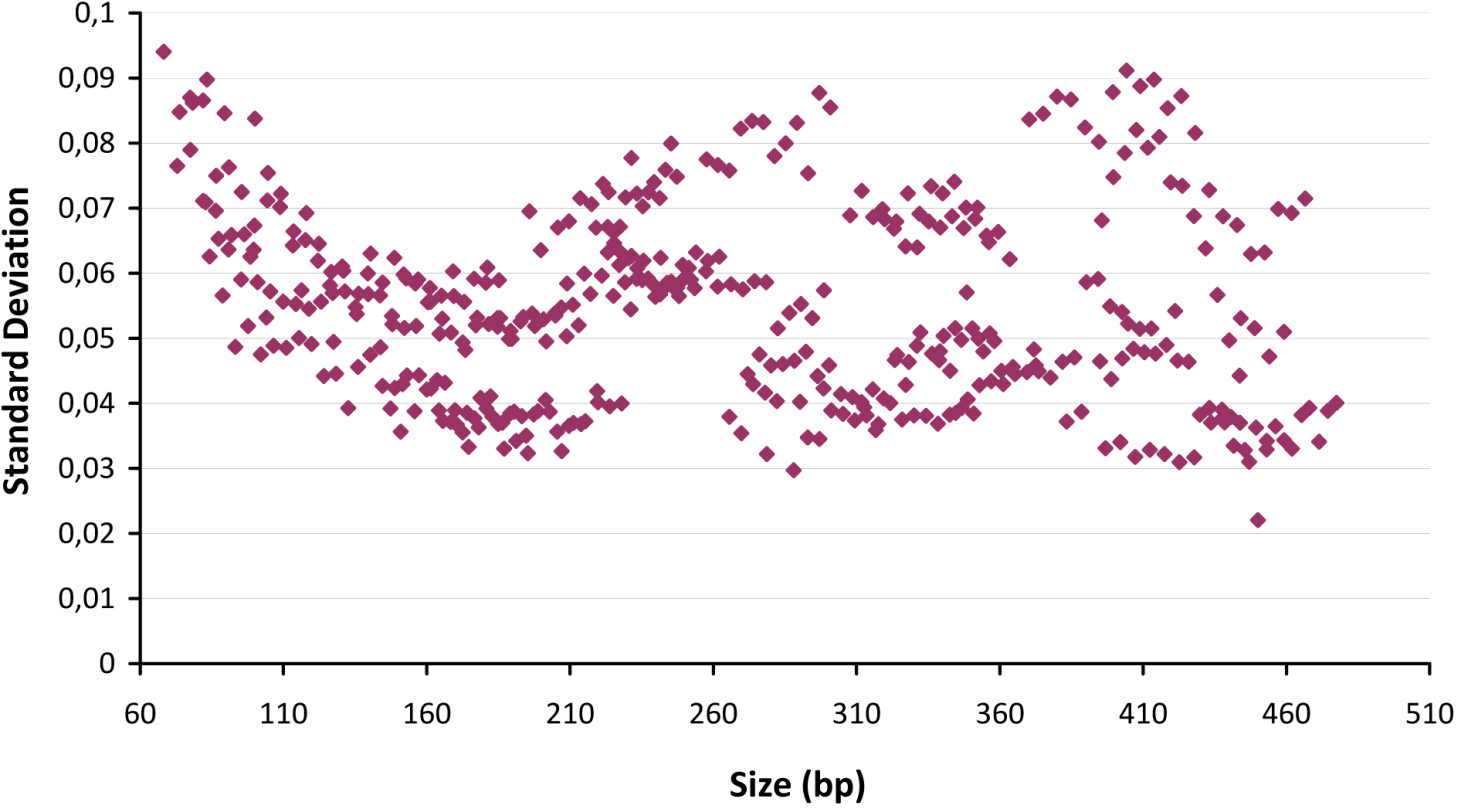
Precision of WEN ILS 500. The average size (bp) of each allele was plotted against the standard deviation observed across 16 ladders run during 5 injections on the same day on Applied Biosystems^®^ 3500 Genetic Analyzer.

The sizes were averaged, and a standard deviation for each allele was plotted against the allele size (Figure 4). The standard deviation had an approximate uniform trendline for all fragment size and did not exceed 0.1 when running on the Applied Biosystems^®^ 3500 Genetic Analyzer. Further, we did not observe statistically significant differences in the sizing precision among different loci and with a confidence interval of 99.7%, the WEN ILS 500 demonstrated the accuracy of sizing over the entire fractionation range for genotyping. These results showed that the precision throughout the assay is adequate to size and distinguish alleles which differ by as little as one base. This high level of precision provides a measure of reliability.

## DISCUSSION

The 27-plex PowerPlex^®^ Fusion 6C System was designed to allow human identification applications including forensic analysis, relationship testing and research use. The system combines common and informative loci to overcome typical challenges faced by laboratories, including low levels of input DNA, sample inhibitors, degradation and mixtures. The PowerPlex^®^ Fusion 6C System includes the 23 loci in the expanded CODIS core loci panel (including SE33), as well as Penta D and Penta E loci for greater discriminatory power, delivering more information in demanding forensic, paternity and relationship-testing cases. Three Y-STRs, two of which are rapidly mutating, were also included to improve mixture interpretation. This evaluation study examined many aspects of kit performance, following SWGDAM validation guidelines. The results of the performed validation studies demonstrated the robustness and reliability of the kit. The amplification chemistry of PowerPlex® Fusion 6C System provided accurate profiles and a wide range of input target DNA. Good results were also obtained from artificially inhibited samples and we demonstrated the potential limitations and the consistency verifying the accuracy of sizing, without statistically significant differences in the sizing precision among different loci. Therefore, profiles typed with this new kit would be of sufficient quality to be uploaded into DNA database and to do new population genetics studies. Comparison with other available amplification kits proves concordance and the reliability of the new primer set has been validated. PowerPlex® Fusion 6C improves the robustness and efficiency of the multiplex STRs system, such as minimizing sample consumption and maximizing informative data output. The greater number of alleles creates greater specificity and generates more data with the same amount of DNA, when compared to previous STR typing kits. Moreover the possibility to obtain Y-STRs profiles in low ratio male to female mixtures is of particular practical case application value. According to the guideline of quality assurance standards for forensic DNA testing laboratories, we carried out an internal validation and demonstrated reliability of PowerPlex® Fusion 6C system. The use of this amplification kit is suitable for the use in future casework samples. Additionally the short amplification time of this kit combined with the automatic CE detection system make it practical for a high-throughput workflow that generates good profile and great output data in less processing time.
