# Supplementary material for: PowerPlex® Fusion 6C System: evaluation study for analysis of casework and database samples
Source: Croat Med J. 2017 Feb;58(1):26–33. doi: 10.3325/cmj.2017.58.26 (PMC5346900; doi:10.3325/cmj.2017.58.26)

Supplemental Figure 1. Sensitivity study of PowerPlex® Fusion 6C System. The graph shows the percent of a full profile of 2800 M DNA above 175 RFU, based on the mean allele count for the two replicate PCRs. The full profile contained 47 alleles total. The standard deviation is also shown.

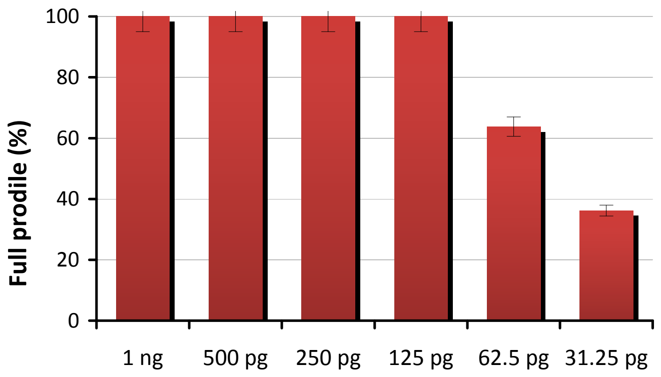

Supplement: Supplementary Figure 1 [file CroatMedJ_58_s001.pdf]
