# Supplementary material for: PowerPlex® Fusion 6C System: evaluation study for analysis of casework and database samples
Source: Croat Med J. 2017 Feb;58(1):26–33. doi: 10.3325/cmj.2017.58.26 (PMC5346900; doi:10.3325/cmj.2017.58.26)

Supplemental Figure 2. Representative inhibited model samples; electropherograms from different inhibited samples (0M, 1M, 2M and 5M of urea) are shown respectively in panels A-D.

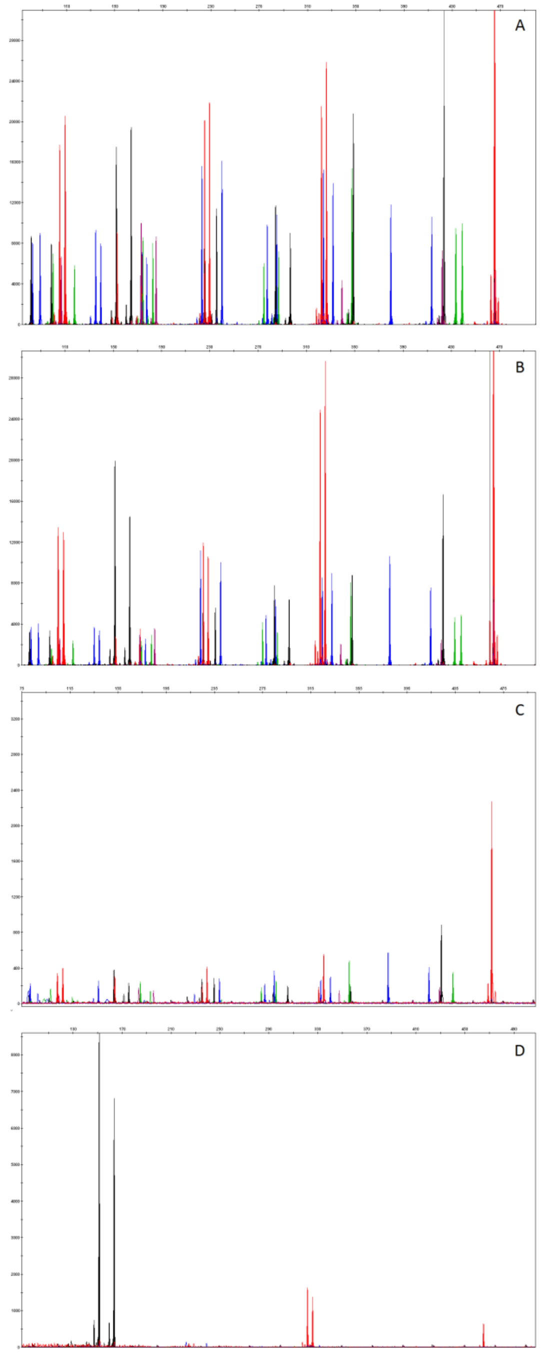

Supplement: Supplementary Figure 2 [file CroatMedJ_58_s002.pdf]
