# Supplementary material for: PowerPlex® Fusion 6C System: evaluation study for analysis of casework and database samples
Source: Croat Med J. 2017 Feb;58(1):26–33. doi: 10.3325/cmj.2017.58.26 (PMC5346900; doi:10.3325/cmj.2017.58.26)

Supplemental Figure 3. Detection of male DNA in a mixture with major female DNA. The electropherogram shows the 1:10 ratio male to female mixture, using the two different human genomic control DNAs (male 2800M and female 9947a, Promega).

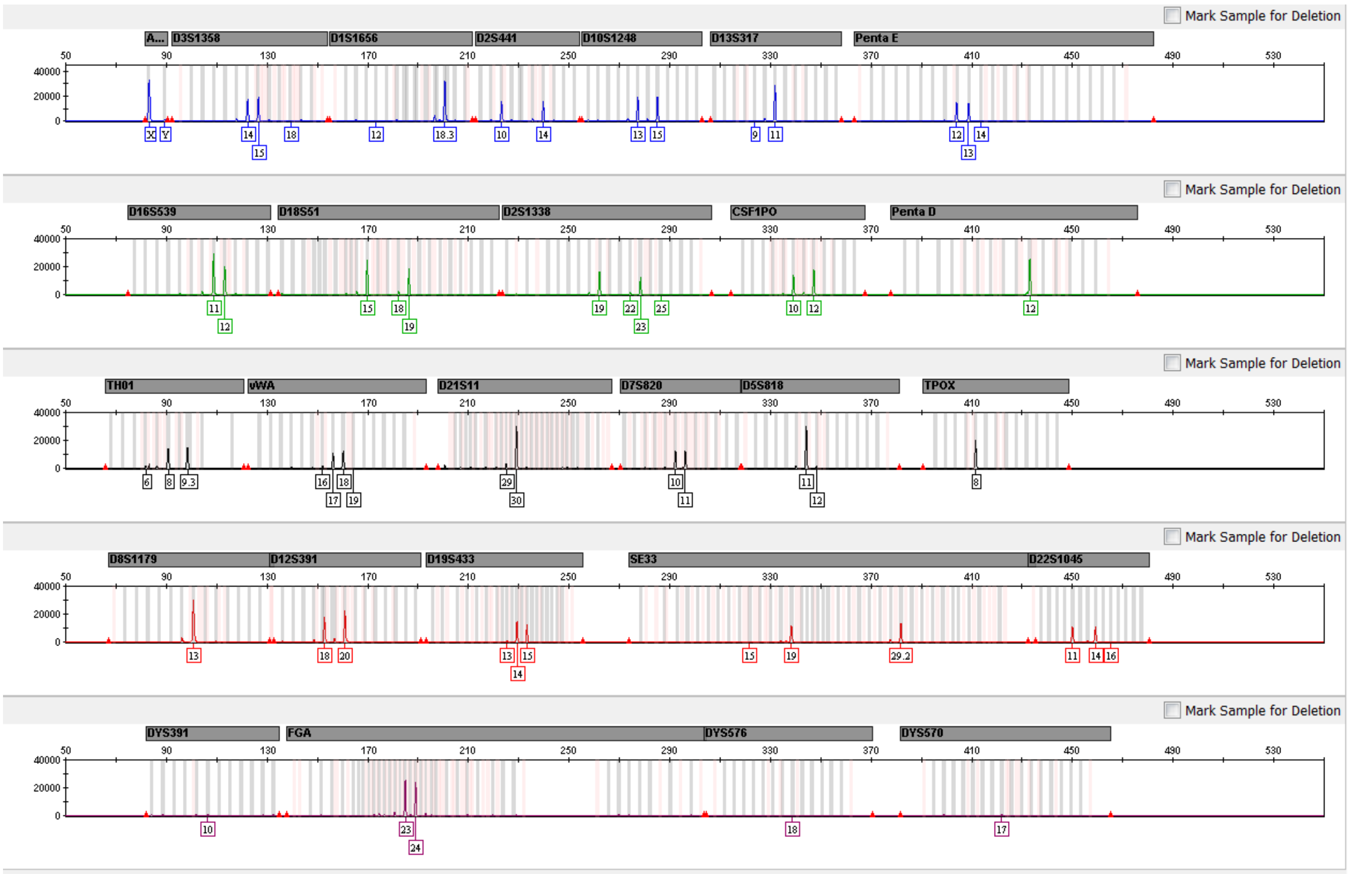

Supplement: Supplementary Figure 3 [file CroatMedJ_58_s003.pdf]
